# Predictors of psychiatric emergency department visits within twelve months post-inpatient psychiatric discharge in Alberta, Canada

**DOI:** 10.1371/journal.pone.0351753

**Published:** 2026-07-08

**Authors:** Hossam Eldin Elgendy, Reham Shalaby, Ernest Owusu, Wanying Mao, Belinda Agyapong, Wes Vuong, Ejemai Eboreime, Nnamdi Nkire, Yifeng Wei, Vincent I. O. Agyapong

**Affiliations:** 1 Department of Psychiatry, University of Alberta, Edmonton, Canada; 2 Alberta Health Services, Edmonton Zone, Alberta, Canada; 3 Department of Psychiatry, Dalhousie University, Halifax, Canada; University of Missouri School of Medicine, UNITED STATES OF AMERICA

## Abstract

**Background:**

Psychiatric emergency department (ED) visits following discharge from inpatient psychiatric care remain a significant challenge for mental health systems and may reflect gaps in continuity of care and community support. While sociodemographic and clinical predictors of recurrent ED utilization have been widely studied, the role of inpatient satisfaction and transitional care interventions in predicting post-discharge psychiatric ED visits remains less clear.

**Objectives:**

This study aimed to examine predictors of psychiatric ED visits within 12 months following discharge from acute psychiatric inpatient units, with particular focus on inpatient satisfaction, prior ED utilization, intervention exposure, sociodemographic characteristics, and clinical factors.

**Methods:**

This observational cohort analysis used data from participants recruited through a pragmatic stepped-wedge transitional care study in Alberta, Canada. A multivariable logistic regression model was conducted to identify predictors of psychiatric ED visits within 12 months post-discharge. Predictor variables included intervention group [treatment as usual (TAU), supportive text messaging (SMS), and supportive text messaging combined with peer support (SMS + PS)], prior ED visits within 6 months before index admission, inpatient satisfaction, sociodemographic variables, and clinical characteristics.

**Results:**

The study included 1,070 participants. Prior psychiatric ED visits within 6 months preceding the index admission emerged as the strongest predictor of post-discharge psychiatric ED utilization. Unemployment and housing instability were also significantly associated with increased likelihood of ED visits within 12 months following discharge. In contrast, inpatient satisfaction, intervention group, gender, ethnicity, relationship status, resilience, wellbeing, depression, and anxiety measures were not independently associated with post-discharge psychiatric ED visits.

**Conclusion:**

Psychiatric ED visits following discharge were primarily associated with prior ED utilization and socioeconomic factors, particularly unemployment and housing instability. Although inpatient satisfaction represents an important component of patient-centered psychiatric care, it was not independently associated with subsequent psychiatric ED visits in this cohort. These findings highlight the importance of addressing structural and social determinants alongside transitional care planning to reduce recurrent psychiatric ED utilization.

## 1. Introduction

The delivery of high-quality inpatient psychiatric services remains a central challenge for mental-health systems worldwide. Individuals admitted for acute psychiatric care often face complex psychosocial risks, and the transition from hospital care to the community is recognized as a particularly vulnerable period [1–3]. Studies consistently show that patients discharged from inpatient psychiatric units exhibit elevated rates of emergency department (ED) visits, hospital readmissions, suicidality, and other adverse outcomes [4–7]. Repeated emergency department visits after an inpatient discharge places a substantial financial and operational burden on hospitals. Psychiatric presentations require specialized assessments and frequently lead to prolonged ED stays due to challenges with placement and bed shortages. This cycle contributes to ED congestion, increases staff workload, and drives up health system costs [8–10]. Moreover, outcomes such as ED visits have been linked to a range of clinical, sociodemographic and service factors (e.g., substance use comorbidity, homelessness at discharge) [5,11]. While much of the available literature focuses on measurable outcomes (e.g., readmission, ED utilization, mortality), there is growing recognition that patient‐reported experience particularly inpatient satisfaction with hospital services, may serve as an upstream indicator of inpatient care quality and may influence post-discharge outcomes [12–14]. In general medical settings, patient satisfaction has been associated with better care engagement, adherence to discharge instructions, reduced acute care use, and improved outcomes. Accordingly, the concept of inpatient satisfaction reflecting patients’ perceptions of the care environment, the therapeutic alliance, discharge processes, and information provision may be particularly salient for psychiatric care, where engagement, trust, and continuity are critical [15,16]. Existing literature reveals that the post-discharge period is characterized by elevated risk, including limited social support, inadequate access to outpatient mental health services, financial inadequacy, and unstable housing status, which contribute to their increased utilization of emergency mental health care services [17,18]. Factors contributing to increased ED utilization following discharge encompass a range of sociodemographic and clinical variables. Patient characteristics such as age, ethnicity, employment status, and housing status have been consistently linked to a higher likelihood of return visits to the ED. A study conducted in Ontario found that being homeless at discharge was associated with nearly double the hazard of an ED visit relative to non-homeless peers [19]. Clinical factors, including primary psychiatric diagnoses, symptom severity at the time of discharge, and prior patterns of healthcare usage, further elevate this risk [20–22]. This study examines predictors of psychiatric emergency department (ED) visits within 12 months following discharge from acute psychiatric inpatient units among participants enrolled in a broader stepped-wedge study evaluating transitional care interventions. The analysis considered multiple potential predictors simultaneously, including study intervention exposure [treatment as usual (TAU), supportive text messaging (SMS), and supportive text messaging combined with peer support (SMS + PS)], inpatient satisfaction, sociodemographic characteristics, prior healthcare utilization, and clinical factors. In addition to exploring whether transitional care interventions were associated with subsequent ED utilization, this study specifically aimed to examine whether patient satisfaction during hospitalization independently predicted post-discharge psychiatric ED visits. The findings are expected to inform healthcare providers and policymakers in refining transitional care strategies, with the goal of reducing pressures on emergency psychiatric services and enhancing patient-centered recovery outcomes.

## 2. Methods

### 2.1. Study design and data collection

This study was conducted in Alberta, Canada. As of July 1, 2023, the provincial population was estimated at 4,695,290 residents, according to the Government of Alberta. Data for this paper includes patients recruited between March 8, 2022, and February 10, 2024. Participants were recruited across all the acute mental health units in three main regions: Edmonton, Calgary, and Grande Prairie, strategically selected for their geographic diversity and participation in a broader regional initiative. The study employed a pragmatic stepped-wedge cluster-randomized design to recruit participants, with the aim to evaluate the effects of supportive text messages alone (Text4Support) and combined with peer support services (PSS) compared to the standard care (TAU) on post-discharge ED utilization among individuals discharged from acute psychiatric inpatient units [23]. Treatment as Usual (TAU) Participants received the standard discharge planning and follow-up services routinely provided by the participating psychiatric units and community mental health programs. Supportive Text Messaging (SMS) Participants in the SMS group received automated supportive text messages through the Text4Support program following discharge from inpatient psychiatric care. Messages were developed collaboratively by psychiatrists, psychologists, and individuals with lived experience of mental illness. The messages were grounded in cognitive behavioral therapy principles and focused on encouragement, coping strategies, recovery, medication adherence, hope, and help-seeking behaviors. Messages were delivered automatically once daily for six months following discharge. The SMS intervention was centrally coordinated through the research program rather than delivered individually by hospital staff. Supportive Text Messaging plus Peer Support (SMS + PS) where In addition to receiving the same daily supportive text messages described above, participants in the SMS + PS group were connected with trained peer support workers with lived experience of mental illness and recovery. Peer support services were provided through community-based peer support programs affiliated with the study. Contacts generally occurred through personal communication, with frequency tailored to participant needs, particularly during the early post-discharge transition period. Peer support focused on emotional support, recovery encouragement, community resource linkage, and assistance with maintaining engagement in outpatient care. A sub-study was also conducted to assess inpatient satisfaction with hospital care for individuals with mental illness following their discharge from acute mental health facilities in Alberta. Recruitment was supported by unit managers, physicians, and nurses at each site, who helped identify eligible participants. Following informed consent via a paper-based form, participants completed two self-administered online surveys using REDCap (Research Electronic Data Capture), a secure web-based platform designed for survey management [24]. The survey instruments attached as supplementary files were developed by the Decision Support Services of the Edmonton Zone, Alberta Health Services (Addiction and Mental Health Program) [25]. The surveys were administered during the participants’ inpatient stay and took approximately 15–20 minutes to complete. Anonymity and confidentiality of responses were emphasized. The first survey collected sociodemographic information, including age, sex, ethnicity, marital status, housing status, and education. Psychiatric diagnoses were initially self-reported by participants and subsequently verified through clinical records at the time of recruitment. The second survey was developed to assess patients’ satisfaction with psychiatric inpatient hospital care. The survey included both quantitative and qualitative questions. For this study, only the quantitative questions were applied. Responses were recorded using a 5-point Likert scale, later consolidated into three categories for analysis: “Yes” (including “Yes definitely” and “Yes to some extent”), “Neutral,” and “No” (including “Not really” and “Definitely not”). Development of the survey instrument was guided by expert consultation, including clinicians, mental health practitioners, and health services researchers, to ensure content validity, relevance, and clarity. The final version was compared against established validated instruments (e.g., HCAHPS, PPE-15) to further support its validity [26,27]. Instrument reliability was evaluated using Cronbach’s alpha, yielding a value of 0.70 in a sample of 1,070 participants, indicating acceptable internal consistency [28,29]. To mitigate response biases such as social desirability, participation was emphasized as voluntary and anonymous, and standardized Likert response scales were employed to reduce external influence on participants’ answers. Participants’ ED visits status within 12 months post-discharge was obtained through Linkage to administrative health records, subject to data-sharing agreements and ethics approval. Also, clinical variables such as level of depression, level of anxiety, resilience, and general well-being were collected at discharge using these validated and standardized scales, Patient Health Questionnaire-9 (PHQ-9) [30,31], Generalized Anxiety Disorder-7 (GAD-7) [32], Brief Resilience Scale-6 (BRS-6) [33], and World Health Organization Five Well-being Index (WHO-5) scale [34], respectively.

### 2.2. Ethics statement

The University of Alberta’s Health Research Ethics Board approved this study (Ref # Pro00111459), and the regional health authority granted further operational approval. Before being included in the study, individuals signed written informed consent forms.

### 2.3. Inclusion and exclusion criteria

The study selection criteria for participants adhered to those outlined in the published study protocol, which aimed to assess the impact of supportive text messages (Text 4 Support) and Peer Support Services (PSS) on individuals with mental illness following their discharge from acute mental health facilities in Alberta [23]. In summary, participants needed to be diagnosed with a mental illness, be at least eighteen years old, scheduled for discharge from an inpatient psychiatric facility, and possess a mobile phone device with an active phone number. The research team collected participants’ phone numbers and healthcare numbers, which served as primary identifiers. Additionally, participants had to be capable of receiving and reading English text messages and have the capacity to provide informed consent.

### 2.4. Outcome measures

The primary outcome examined in this study was psychiatric emergency department (ED) visits within 12 months following discharge from the index psychiatric inpatient admission. The study aimed to evaluate whether intervention exposure (TAU, SMS, SMS + PS), inpatient satisfaction, prior psychiatric healthcare utilization, sociodemographic characteristics, and clinical variables were associated with the likelihood of post-discharge psychiatric ED visits.

### 2.5. Statistical analysis

Data were analyzed using SPSS version 25 for Mac (IBM Corp., USA) [35]. Descriptive statistics summarized participants’ sociodemographic and clinical characteristics in relation to psychiatric emergency department visits within 12 months preceding index admission. A multiple binary logistic regression model was conducted to identify significant predictors of emergency visits within the 12-month post-discharge period [35]. Predictor variables entered into the model included, study clusters (SMS, SMS+Peer support, and TAU), prior emergency visits, satisfaction with inpatient care, age, gender, ethnicity, educational attainment, employment status, housing situation, relationship status, and several mental health screening measures (PHQ-9, GAD-7, BRS-5 and theWHO-5,). Odds ratios (ORs) with corresponding confidence intervals (C.I.) were calculated to evaluate the strength and statistical significance of these predictors. Study intervention group (TAU, SMS, SMS + PS) was included in the regression model as one of several potential predictor variables rather than as the primary exposure of an intervention-effectiveness analysis.

## 3. Results

Table 1 presents the univariate analysis examining factors associated with participants’ ED visit in the 12 months before their index admission. Across the full sample of 1,070 participants, most were between 25 years old or younger (395, 36.9%), with slightly more females than males (586 54.8%). The majority identified as Caucasian (586, 54.8%), held a high school diploma (554, 51.8%), and were single at the time of data collection (633, 59.2%). Over half of the participants were unemployed (568, 53.1%), and most lived with family or friends or in rented accommodation, with a smaller proportion owning their homes (211, 19.7%). More than half of the sample demonstrated low resilience (603, 56.4%). Most the participants were very satisfied or satisfied with their prior inpatient experience (831, 88.9%). Wellbeing, depression, and anxiety symptom categories were relatively evenly distributed. Overall, 40% of participants recorded an ED visit within 12 months post–discharge, and participants were distributed across the three study clusters (SMS, SMS + PS, and TAU), with the largest proportion assigned to TAU (432, 39.2%).

**Table 1:** Distribution of demographic and clinical characteristics of the participants against the 12- month prior ED visit before index admission.

Variables	No prior ED visits	Prior ED visits	Total n = 1070
**Age**			
≤ 25 y	24 (6.1)	371 (93.9)	395 (36.9)
26-40y	21 (5.8)	344 (94.2)	365 (34.1)
> 40y	22 (7.1)	288 (92.9)	310 (29.0)
**Gender**			
Males	27 (6.0)	425 (94.0)	452 (42.2)
Females	36 (6.1)	550 (93.9)	586 (54.8)
Other	4 (12.5)	28 (87.5)	32 (3.0)
**Ethnicity**			
Caucasian	44 (6.7)	617 (93.3)	661 (61.8)
Indigenous	3 (3.0)	95 (97.0)	98 (9.2)
Black	8 (6.9)	108 (93.1)	116 (10.8)
Asian	5 (4.1)	116 (95.1)	121 (11.3)
Mixed/Other	7 (9.5)	67 (90.5)	74 (6.9)
**Education level**			
Less than High School	3 (7.7)	36 (92.3)	39 (3.6)
High School Diploma	34 (6.1)	520 (93.9)	554 (51.8)
Post-secondary Education	27 (6.1)	416 (93.9)	443 (41.4)
Prefer not to say	3 (8.8)	31 (91.2)	34 (3.2)
**Relationship status**			
Married/Partnered/Common-Law	20 (6.4)	291 (93.6)	311 (29.1)
Single	40 (6.3)	593 (93.7)	633 (59.2)
Separated or Divorced	3 (3.6)	80 (96.4)	83 (7.8)
Widowed	0 (0.0)	12 (100)	12 (1.1)
Prefer not to say	4 (12.9)	27 (87.1)	31 (2.9)
**Employment status**			
Employed	18 (5.7)	299 (94.3)	317 (29.6)
Unemployed	30 (5.3)	538 (94.7)	568 (53.1)
Student	9 (10.3)	78 (89.7)	87 (8.1)
Retired	6 (9.5)	57 (90.5)	63 (5.9)
Other	4 (11.4)	31 (88.6)	35 (3.3)
**Housing status**			
Own Home	15 (7.1)	196 (92.9)	211 (19.7)
Rented Accommodation	19 (5.5)	326 (94.5)	345 (32.3)
Live with Family or Friends	31 (6.9)	416 (93.9)	447 (41.8)
Other (Couch Surfing, Shelter, Street)	1 (1.5)	65 (98.5)	66 (6.2)
**Inpatient Satisfaction**			
Very satisfied (9-10)	26 (7.6)	314(92.4)	340 (36.4)
Satisfied (6-8)	24 (4.9)	467 (95.1)	491 (52.5)
Not Satisfied (<6)	5 (4.8)	99 (95.2)	104 ((11.1)
**Brief Resilience Scale Category (BRS)**			
Normal to High Resilience	24 (5.2)	442 (94.8)	466 (43.6)
Low Resilience	42 (7)	561 (93)	603 (56.4)
**WHO-5 Category**			
Good Wellbeing	37 (6.8)	511 (93.2)	548 (51.3)
Poor Wellbeing	29 (5.6)	491 (94.4)	520 (48.7)
**Depression Category (PHQ-9)**			
Unlikely Depression	34 (7.4)	423 (92.6)	457 (42.8)
Likely Depression	32 (5.2)	579 (94.8)	611 (57.2)
**Anxiety Category (GAD-7)**			
Unlikely anxiety	42 (6.2)	633 (93.8)	675 (63.6)
Likely anxiety	22 (5.7)	365 (94.3)	387 (36.4)
**ED visits within 12 months (post-discharge)**			
No	61 (9.2)	600 (90.8)	661 (60)
Yes	9 (2)	431 (98)	440 (40)
**Cluster**			
SMS	16 (5.2)	293 (94.8)	309 (28.1)
SMS + PS	38 (10.6)	322 (89.8)	360 (32.7)
TAU	16 (3.7)	416 (96.3)	432 (39.2)

Table 2 illustrates the results of a binary logistic regression analysis predicting the likelihood of ED visits within twelve months following discharge. Fourteen independent variables were entered into the model, including age category, gender, ethnicity, education, current relationship status, employment status, current housing status, inpatient satisfaction, mental health diagnosis, Brief Resilience Scale (BRS) category, WHO-5 wellbeing category, PHQ-9 depression category, GAD-7 anxiety category, study cluster, and admission status twelve months prior to the index admission. The overall regression model was statistically significant, Χ²(31, N = 929) = 82.24, p = < .001, indicating that the predictors, taken together, reliably distinguished between participants who were readmitted and those who were not. The model explained between 8.5% (Cox & Snell R²) and 11.4% (Nagelkerke R²) of the variance in ED visits status and correctly classified 76.0% of cases. In this analysis, none of the key participant characteristics, such as study group, satisfaction level, age, gender, ethnicity, education, relationship status, resilience, wellbeing, depression, or anxiety, were significantly associated with ED visits within the following 12 months. The confidence intervals for all odds ratios crossed unity, indicating that these factors did not independently contribute to predicting ED visits 12 months post-discharge in the adjusted model. In contrast, employment and housing status emerged as significant predictors of future ED utilization. Unemployed Participants had significantly higher odds of returning to the ED (OR = 1.57, 95% CI: 1.11–2.21) compared to those who were employed. Housing instability showed a similarly strong association; individuals living in shelters, couch-surfing situations, or on the street were more than twice as likely to visit the ED (OR = 2.60, 95% CI: 1.29–5.22) relative to homeowners. Prior ED utilization stood out as the strongest predictor in the model. Participants who had an ED visit in the six months before the index admission had more than triple the odds of returning within the year following their hospital discharge (OR = 3.72, 95% CI: 1.82–7.63).

**Table 2:** Multivariate logistic regression model predicting the likelihood of the ED visits within 12 months post-discharge among study participants.

Characteristics	B	SE	Wald	df	P Value	Odds ratio	95%C.I.	
							Upper	Lower
**Study Cluster**								
SMS			2.228	2	.328			
SMS + PS	−.232	.190	1.485	1	.223	.793	.546	1.152
TAU	−.233	.171	1.859	1	.173	.792	.566	1.108
**Satisfaction Cat**.								
Very Satisfied			1.358	2	.507			
Satisfied	.082	.155	.281	1	.596	1.086	.801	1.472
Not Satisfied	.282	.242	1.351	1	.245	1.326	.824	2.132
**Age Cat.**								
≤ 25 years			2.576	2	.276			
26–40 years	−.109	.186	.342	1	.559	.897	.623	1.291
> 40 years	−.373	.238	2.462	1	.117	.689	.432	1.097
**Gender Cat**.								
Male			1.111	2	.574			
Female	−.099	.150	.441	1	.507	.905	.675	1.214
Other	−.410	.437	.881	1	.348	.664	.282	1.562
**Ethnicity Cat.**								
Caucasians			7.141	4	.129			
Indigenous People	.138	.245	.316	1	.574	1.148	.710	1.855
Black People	−.342	.244	1.964	1	.161	.710	.440	1.146
Asians	.159	.235	.456	1	.500	1.172	.739	1.857
Other	−.600	.306	3.839	1	.050	.549	.301	1.000
**Education Cat.**								
Less than High School			5.508	3	.138			
High School Diploma	−.451	.375	1.442	1	.230	.637	.305	1.330
Post-Secondary Educ	−.739	.383	3.712	1	.054	.478	.225	1.013
Other	−.660	.543	1.477	1	.224	.517	.178	1.498
**Current relationship.**								
Partnered/Married/Common law.			5.335	4	.255			
Single	−.099	.179	.304	1	.582	.906	.637	1.288
Separated/Divorced	−.041	.302	.019	1	.891	.960	.531	1.735
Widowed	−.930	.851	1.194	1	.274	.395	.075	2.091
Other	.904	.515	3.084	1	.079	2.470	.900	6.777
**Current employment.**								
Employed			10.782	4	.029			
Unemployed	.450	.175	6.644	1	.010	1.569	1.114	2.209
Student	−.013	.305	.002	1	.966	.987	.542	1.796
Retired	−.201	.393	.262	1	.609	.818	.379	1.766
Other	−.133	.471	.080	1	.778	.875	.348	2.203
**Current housing**								
Own Home			10.819	3	.013			
Rented Accommodation	.390	.224	3.046	1	.081	1.477	.953	2.290
Live with Family/Friends	.074	.245	.091	1	.763	1.077	.666	1.739
Couch/Shelter/Street/Other	.954	.357	7.143	1	.008	2.595	1.289	5.222
**6 Months prior- ED Visits**	1.314	.366	12.859	1	.000	3.722	1.815	7.633
**BRS Cat.**								
Normal to High Resilience								
Low Resilience	−.143	.158	.815	1	.367	.867	.636	1.182
**WHO Cat.**								
Good Wellbeing								
Poor Wellbeing	.054	.168	.101	1	.750	1.055	.758	1.468
**PHQ Cat**.								
Likely Depression								
Unlikely Depression	.166	.176	.887	1	.346	1.180	.836	1.666
**GAD Cat.**								
Likely Anxiety								
Unlikely Anxiety	−.139	.176	.626	1	.429	.870	.616	1.228
Constant	−1.146	.595	3.705	1	.054	.318		

## 4. Discussion

The present observational analysis in this study examined the association between multiple factors, including intervention exposure, inpatient satisfaction, sociodemographic characteristics, prior healthcare utilization and clinical variables, and psychiatric ED visits within 12 months following discharge from psychiatric inpatient care. The findings suggest that socioeconomic and structural factors had a greater influence on post-discharge ED utilization than most clinical or demographic characteristics which demonstrated little predictive power. The multivariate model revealed that only unemployment, housing instability, and previous ED use independently predicted future ED visits within a year post-discharge, despite the univariate analysis showing variability in ED visits across age, ethnicity, employment, and housing categories. This pattern highlights the complex relationship between healthcare utilization and social vulnerability, which is in line with a large body of research highlighting the importance of social determinants in influencing mental health outcomes [36–41]. Unemployment emerged as a significant predictor of subsequent ED visits, aligning with previous research demonstrating that economic constraint is strongly associated with increased acute mental-health service use [39,42,43]. Economic instability may exacerbate psychiatric symptoms, limit access to primary or community-based services, and diminish individuals’ capacity to engage in preventive care [43,44]. Similarly, housing instability was one of the strongest predictors of ED return visits in the present study. This is consistent with international and Canadian evidence showing that individuals who are homeless or unstably housed experience disproportionately higher rates of ED visits and hospitalizations [37,45–47]. An Ontario cohort study found that psychiatric patients who were homeless at discharge had nearly double the hazard of a mental health-related ED visit within 30 days compared with non-homeless patients, suggesting that housing instability may reduce treatment continuity, intensify stressors, and limit the ability to adhere to discharge plans, thereby increasing the likelihood of crisis presentations [19]. Prior ED use was the strongest predictor of subsequent ED utilization, suggesting a pattern of recurrent service use that may reflect unresolved clinical needs, inadequate transition support, or unmet psychosocial challenges. This finding is widely supported in the literature, with past ED use consistently identified as one of the most reliable predictors of future acute-care episodes across psychiatric and general medical populations [48–51]. Such patterns reinforce the importance of targeted interventions for high-utilizing groups, potentially including intensive case management, strengthened outpatient follow-up, and enhanced community supports. The present study evaluated psychiatric ED utilization across a 12-month follow-up period to capture broader patterns of post-discharge acute-care use. However, recurrent ED presentations may be concentrated within the earlier months following discharge, which could represent a period of heightened clinical and social vulnerability. Future studies examining earlier timing and distribution of ED presentations, may help identify critical windows for targeted transitional interventions and relapse prevention strategies. Contrary to expectations, neither psychiatric diagnosis, clinical characteristics (depression, anxiety, wellbeing), resilience, nor inpatient satisfaction predicted post-discharge ED visits in the adjusted model. While patient-reported experience has been hypothesized to influence post-discharge engagement and outcomes, evidence remains mixed. Some studies have found associations between poor satisfaction and increased ED visits risk [52–54], whereas others, including a large multicenter prospective study, report no independent effect after adjusting for clinical and sociodemographic variables [55,56]. Our findings add to the latter group, suggesting that socioeconomic disadvantages may predominate the influence of individual perceptions and clinical characteristics in driving acute care utilization among psychiatric populations. This study adds to the existing literature by simultaneously examining inpatient satisfaction, intervention exposure, sociodemographic factors, prior healthcare utilization, and clinical variables as predictors of psychiatric ED visits following discharge. While factors such as unemployment, housing instability, and prior ED use have been previously identified, fewer studies have evaluated these variables alongside patient-reported inpatient satisfaction within the same adjusted model and within a real-world transitional care setting. Additionally, the nonsignificant association between inpatient satisfaction and subsequent ED visits contributes to the limited and mixed evidence regarding the independent role of patient experience in predicting acute psychiatric service utilization after discharge. However, the identified predictors reflect modifiable social conditions that may be addressed through policymakers and service interventions. For example, enhancing access to stable housing, supported employment, and coordinated transitional care services may reduce repeated ED presentations and improve continuity of care [37,57–60]. All together, these findings underscore the need for mental health systems that prioritize structural and social supports in transitional care planning. While clinical and demographic factors contribute to understanding patient needs, it is the broader social environment, particularly housing stability, economic security, and prior care patterns, that appears to govern post-discharge ED visit patterns. Interventions that address these determinants may offer more substantial and sustained reductions in acute-care utilization than those focused solely on clinical or patient-experience variables. Although this study contributes to the international literature on psychiatric emergency department utilization, its findings should be interpreted within the context of the Canadian healthcare system. Canada’s publicly funded healthcare model reduces financial barriers to accessing both inpatient and emergency psychiatric services, which may influence patterns of ED utilization compared to countries with privatized or insurance-based systems. In such settings, cost-related barriers may delay care-seeking or shift utilization toward alternative services, potentially altering the relative importance of predictors identified in this study.

### 4.1. Limitations

Several limitations should be considered when interpreting the findings of this study. The study sample might not fully represent the wider population of psychiatric patients, especially those who are not engaged with services or harder to reach. Key variables such as patient satisfaction and clinical characteristics were derived from self-report measures, which may be subject to recall bias or social desirability effects and could affect the accuracy of the data. Additionally, since the study took place within one healthcare system and geographic area, the findings might not apply to other settings with different patient populations, resources, or care models, which limits generalizability. Furthermore, the use of broad diagnostic categories may obscure important distinctions between specific psychiatric conditions and their unique associations with recurrent ED visits. Other potentially influential factors, such as medication adherence, quality of social support, comorbid medical conditions, and access to outpatient follow-up, were not captured in the dataset. Despite these constraints, the study provides valuable insights into the sociodemographic and clinical determinants of repeated psychiatric ED visits and highlights key areas for future investigation and targeted intervention.

## 5. Conclusion

The current study highlights that socioeconomic factors and prior psychiatric healthcare utilization are the primary determinants of emergency department visits following psychiatric admission. Unemployment, unstable housing, and a history of recent ED visits significantly increased the likelihood of recurrent ED visits within 12 months post-discharge. In contrast, sociodemographic and clinical characteristics, including age, gender, ethnicity, education, relationship status, psychiatric diagnosis, symptom severity, resilience, wellbeing, and inpatient satisfaction, did not independently predict ED use post-discharge from psychiatric units. These findings reveal the important role of social determinants and prior service use in shaping post-discharge ED utilization and suggest that interventions targeting economic stability, housing support, and continuity of care may be crucial in reducing recurrent ED use among psychiatric patients.

## Supporting information

S1 File Servey satisfaction reducing readmission. (PDF)
