# Supplementary material for: Predictors of psychiatric emergency department visits within twelve months post-inpatient psychiatric discharge in Alberta, Canada
Source: PLoS One. 2026 Jul 8;21(7):e0351753. doi: 10.1371/journal.pone.0351753 (PMC13345297; doi:10.1371/journal.pone.0351753)
Supplement: S1 File — (PDF) [file pone.0351753.s001.pdf]

satisfactory

Thank you for completing this survey. The purpose of this survey is to receive feedback on your experience related to your current inpatient treatment. The information you provide will help us improve the service for you and others in the future.

This survey will take about 5 minutes to complete.Your participation is voluntary.Responses are anonymousThe information you provide will not be used to identify you – results will be reported as a group.We recommend that surveys be completed when connected to Wi-Fi.Completing the survey means that you agree to take part in the evaluation.

Please enter the mobile number (e.g., 780-123-1234) that you would like to receive text message communication from the research team

____

Which Hospital are you currently or did you recently receive inpatient treatment?

- ◯ Edmonton
- ◯ Calgary
- ◯ Grande Prairie

Edmonton

- ◯ Alberta Hospital Edmonton
- ◯ Royal Alexandra Hospital
- ◯ University of Alberta Hospital
- ◯ Grey Nuns Community Hospital
- ◯ Misericordia Community Hospital

Calgary

- ◯ Foothills Hospital
- ◯ Peter Lougheed Center
- ◯ Rockyview Hospital
- ◯ South Heath Hospital

How long have you stayed in the mental health unit during the current hospital admission?

- ◯ Less than one week
- ◯ One to two weeks
- ◯ More than 2 weeks but less than 4 weeks
- ◯ 4 weeks to 8 weeks
- ◯ More than 8 weeks

Please choose an option that best describes your experience during this admission.

	Yes definitely	Yes to some extent	Neutral	Not really	Definitely not
Did you feel welcome?	◯	◯	◯	◯	◯
Was the hospital room you stayed in comfortable?	◯	◯	◯	◯	◯
Did you receive consistent information about your treatment and care from staff?	◯	◯	◯	◯	◯
Did you feel the staff really listened to you?	◯	◯	◯	◯	◯
Did you feel the staff understood your needs and concerns?	◯	◯	◯	◯	◯
Did staff deal with your needs and concerns?	◯	◯	◯	◯	◯
Did you feel the staff were genuine?	◯	◯	◯	◯	◯
Did you find the staff knowledgeable?	◯	◯	◯	◯	◯
Did you feel that you were treated with dignity and respect?	◯	◯	◯	◯	◯
Were your preferences and values respected in your care (e.g., cultural, spiritual, gender, etc.)?	◯	◯	◯	◯	◯

What was your involvement in the development of your Care Plan?

◯ I was involved in the development of my Care Plan
◯ I was not involved in the development of my care plan but I reviewed and approved it
◯ I was not involved in the development of my care plan and I have not reviewed my care plan
◯ I do not know if I have a Care Plan

How often have you reviewed your Care Plan with staff?

◯ At least weekly
◯ Less than weekly
◯ Never

Please choose the best option which describes the Personalization and Effectiveness of the Treatment received.

	Yes definitely	Yes to some extent	Neutral	Not really	Definitely not
Did you feel you were given enough education/information about your condition or issue?	◯	◯	◯	◯	◯
Were you given options or choices for your treatment and care?	◯	◯	◯	◯	◯
Was your treatment and care personalized to meet your needs?	◯	◯	◯	◯	◯
Did you find the treatment and care you received helpful?	◯	◯	◯	◯	◯
Did you feel your treatment and care helped reduce your symptoms and/or issues?	◯	◯	◯	◯	◯
Did you feel you were involved enough in decisions about your treatment and care?	◯	◯	◯	◯	◯

Who primarily supported you over the course of your inpatient care? Please select only one of the below options

◯ Parent(s)
◯ Sibling(s)
◯ Partner(s)
◯ Child/Children
◯ Friend(s)
◯ Counsellor/Therapist
◯ Social Worker
◯ Family Doctor
◯ Psychiatrist
◯ AHS Service(s)
◯ Non-AHS Service(s)
◯ Other

Please specify ______________________________

Please specify ______________________________

Please specify ______________________________

Please choose one option

	Yes definitely	Yes to some extent	Neutral	Not really	Definitely not
Did staff include your family or someone close to you in your care as much as you wanted?	◯	◯	◯	◯	◯
Were you given enough time to talk about your condition or issue with staff?	◯	◯	◯	◯	◯
Did staff tell you about the other services and supports available to you during treatment if you needed them (e.g, physical health needs, child/family care, financial, housing, etc.)?	◯	◯	◯	◯	◯
Did staff help you identify where to get support after you leave the service (e.g., peer support, crisis management, etc.)?	◯	◯	◯	◯	◯
Did your care appear to be well coordinated among all staff at this service?	◯	◯	◯	◯	◯

Did you face any barriers when accessing this inpatient service?

◯ Yes
◯ No

Please specify

____

Overall, how satisfied are you with the service you received?

◯ Very satisfied
◯ Mostly satisfied
◯ Mixed
◯ Mostly dissatisfied
◯ Very dissatisfied

We want to know the overall rating of your care and treatment. Using any number from 1 to 10, where 1 is the worst possible care and 10 is the best possible care, how would you rate your care and treatment at this program/service?

Worst possible care — Best possible care

(Place a mark on the scale above)

What did you like best about the service you received during your stay in the hospital?

____

What would you like to change about the service you received in the hospital?

____

Please add additional comments, suggestions, or questions here

____
